# Functionalised Sodium–Carboxymethylcellulose–Collagen Bioactive Bilayer as an Acellular Skin Substitute for Future Use in Diabetic Wound Management: The Evaluation of Physicochemical, Cell Viability, and Antibacterial Effects

**DOI:** 10.3390/polym16162252

**Published:** 2024-08-08

**Authors:** Maheswary Thambirajoo, Nur Izzah Md Fadilah, Manira Maarof, Yogeswaran Lokanathan, Mohd Ambri Mohamed, Sarani Zakaria, Ruszymah Bt Hj Idrus, Mh Busra Fauzi

**Affiliations:** 1 Department of Tissue Engineering and Regenerative Medicine, Faculty of Medicine, Universiti Kebangsaan Malaysia, Kuala Lumpur 56000, Malaysia; mahe_meera@yahoo.com (M.T.); izzahfadilah@ukm.edu.my (N.I.M.F.); manira@ukm.edu.my (M.M.); lyoges@ppukm.ukm.edu.my (Y.L.); ruszyidrus@gmail.com (R.B.H.I.); 2 Advance Bioactive Materials-Cells UKM Research Group, Universiti Kebangsaan Malaysia, Bandar Baru Bangi 43600, Malaysia; 3 Institute of Microengineering and Nanoelectronics (IMEN), Universiti Kebangsaan Malaysia (UKM), Bandar Baru Bangi 43600, Malaysia; ambri@ukm.edu.my; 4 Materials Science Program, Department of Applied Physics, Faculty of Science and Technology, Universiti Kebangsaan Malaysia, Bandar Baru Bangi 43600, Malaysia; szakaria@ukm.edu.my

**Keywords:** diabetes, wound regeneration, skin pathogens, skin substitute, Na-CMC bilayer scaffold

## Abstract

The wound healing mechanism is dynamic and well-orchestrated; yet, it is a complicated process. The hallmark of wound healing is to promote wound regeneration in less time without invading skin pathogens at the injury site. This study developed a sodium–carboxymethylcellulose (Na-CMC) bilayer scaffold that was later integrated with silver nanoparticles/graphene quantum dot nanoparticles (AgNPs/GQDs) as an acellular skin substitute for future use in diabetic wounds. The bilayer scaffold was prepared by layering the Na-CMC gauze onto the ovine tendon collagen type 1 (OTC-1). The bilayer scaffold was post-crosslinked with 0.1% (*w*/*v*) genipin (GNP) as a natural crosslinking agent. The physical and chemical characteristics of the bilayer scaffold were evaluated. The results demonstrate that crosslinked (CL) groups exhibited a high-water absorption capacity (>1000%) and an ideal water vapour evaporation rate (2000 g/m^2^ h) with a lower biodegradation rate and good hydrophilicity, compression, resilience, and porosity than the non-crosslinked (NC) groups. The minimum inhibitory concentration (MIC) of AgNPs/GQDs presented some bactericidal effects against Gram-positive and Gram-negative bacteria. The cytotoxicity tests on bilayer scaffolds demonstrated good cell viability for human epidermal keratinocytes (HEKs) and human dermal fibroblasts (HDFs). Therefore, the Na-CMC bilayer scaffold could be a potential candidate for future diabetic wound care.

## 1. Introduction

Traditionally, gauze is one of the preferred wound dressings applied to wrap the wound from microbial infections and protect the wound from environmental irritants, such as dust or dirt [1]. Carboxymethyl cellulose (CMC) is a type of anionic, water-soluble polysaccharide and a derivative of cellulose that possesses non-immunogenicity and high water solubility and is biodegradable, biocompatible, and cost-effective [2]. Due to its excellent properties, CMC is considered a promising candidate that can be blended with other substances, such as polymers, hydrogels, and many more, to produce a good biomaterial in the tissue engineering field [3]. CMC-based biomaterials have a good affinity with water polymers like polyethylene glycol, high wettability rate, and increased tensile strength and are suitable for skin, bone, and mucous membranes [4,5]. Among the different chemicals that are made from salt, sodium CMC (Na-CMC) and calcium CMC (Ca-CMC) are generally used in alleviating skin wound healing. Na-CMC was found to be adaptable to the native skin and acts as a barrier preventing pathogens from entering the broken skin [6]. Besides wound applications, beauty products, and manufacturing drugs or medicines, Na-CMC was found to have an essential role in surgery by hampering post-operative adhesion and reducing the formation of epidural scarring [7].

Various methods have been employed in skin wound treatments, including artificial skins, allografts, xenografts, wound dressing, etc., but despite accelerating wound healing, these treatments have some drawbacks. For instance, allografts cause graft rejection after implantation over a long period of time, and wound dressing does not have bioactive molecules to induce wound closure. Considering this situation, opting for different therapeutic techniques is the best choice to overcome the limitations of the abovementioned methods [8,9]. Collagen type I is the major constituent that forms 70% of the skin layer. It consists of an extracellular matrix (ECM) component that provides support to integumentary cells. In normal wound healing, along with degradation products, collagen modulates the secretion of cytokines and growth factors that are needed for tissue repair, the stimulation of new tissue growth, autolytic debridement, angiogenesis, cell migration, proliferation, and re-epithelialisation [10]. Though collagen can be extracted from animal skins of different origin, porcine and bovine skins are highly preferred. However, porcine and bovine collagens are limited due to cultural and religious concern, especially porcine for Muslims and Jews, while bovine for Hinduism and Buddhism [11,12]. Other than religious issues, porcine and bovine collagens are prohibited from being used in research as they pose a risk of transmitting bovine spongiform encephalopathy (BSE) and transmissible spongiform encephalopathy (TSE) to humans [13]. In recent years, ovine tendon collagen type 1 (OTC-1) from sheep has gained more popularity in tissue engineering as it offers a higher yield; possesses good skin cell–scaffold interaction, biodegradability, and non-immunogenicity; and can be fabricated into various scaffolds, such as sponges, nanofibers, gels, films, and sheets, to treat complicated wounds, such as chronic wounds. Many studies have been conducted by using OTC-1 for skin treatments in murine models, and the outcomes showed a good improvement in wound recovery in those animals [8,9].

GNP is a natural crosslinker obtained from the fruits of *Gardenia jasminoides*. GNP not only helps to increase the stiffness of the collagen scaffolds but also exhibits a low cytotoxicity, which is safe to be used in biomaterials [14,15]. GNP can be crosslinked with several types of collagen bilayer scaffolds, like hydrogels, gelatine, chitosan, biological samples, and natural polymers [16]. Apart from tissue engineering and wound dressing, GNP has been largely explored in drug delivery systems for certain medical conditions, such as thrombosis, inflammatory reaction, tumour progression, and diabetes, as well as in some cell studies, including angiogenesis and the growth of nervous tissue [17]. The crosslinking between the amino group of collagens and GNP is based on two steps. The first step is the nucleophilic attack of the silane amino group against the C3 carbon atom of the GNP to develop a heterocyclic compound of GNP. The compound is formed after GNP reacts with free amino groups, such as lysine, hydroxyl lysine, and arginine. This then leads to the opening of the dihydropyran ring and the development of an intermediate aldehyde group. The second step involves the attack by the aldehyde group from secondary amine, which finally seals the ring that was formed earlier [18,19]. On the other hand, citric acid (CA) is a natural organic compound that has three carboxylic groups and is widely used in food processing and has been approved by the Food and Drug Administration (FDA) for its safe use. CA is a weak acid and harmless; therefore, it has become the best choice to be utilised as a crosslinker in polymers such as CMC and poly (ethylene oxide) (PEO). The mechanism of CA crosslinking is by binding the hydroxyl groups of the polysaccharides with the long chain of carbohydrate through a covalent intermolecular di-ester bond, which enhances the stability of the polysaccharide construction [20].

Several studies have evaluated cellulose–collagen-based scaffolds, particularly CMC/Col, and their modifications in the biomedical field focusing on skin engineering [4]. To mention a few, a group of researchers used cellulose and collagen as their primary materials in wound dressing [3,21]. They concluded that the wound size became small within 28 days, and some of the wound inhibitory enzymes, like elastase, plasmin, and gelatinase, were detected in lower quantities [7]. In a similar study, Loh and colleagues proposed the use of an oxidised regenerative cellulose ORC/collagen/silver dressing aligned with negative pressure wound therapy (NPWT) to treat chronic wounds mainly DFU as an alternative for reconstructive flap surgery. According to them, this combination of wound dressing is quite intriguing as it accelerated the wound closure in a minimal time and enhanced the antibacterial function upon testing on 37 patients who had been diagnosed with various comorbidities [22]. There are also other studies that have used similar designs of wound dressing either as a single layer or double layer consisting of a cellulose/collagen bilayer that can mimic the function of the native skin based on the objectives of the studies being conducted. Furthermore, a group of researchers fabricated wound dressings by using alginate/hyaluronic acid/collagen with antimicrobial peptides (AMPs) to treat bacterially infected wounds [23], while another group prepared bilayer-spongy structures with cellulose acetate nanofibers and Ciprofloxacin on top layers and collagens at the bottom, contained keratinocytes and fibroblasts for skin wound regeneration [21]. Kacvinská and colleagues used pure collagen (Col) mixed with Na-CMC and col with oxidised cellulose (OC) to verify the efficiency of these biomaterials for soft tissue approaches [24]. Cemile and colleagues produced bilayer matrices composed of collagen sponge as the epidermis layers and Na-CMC sponge as the dermis layers to treat the chronic wounds [7]. 

Diabetes mellitus (DM) is a group of common endocrine diseases caused by defective insulin secretion; insulin resistance leads to sustained high blood sugar levels, a condition known as hyperglycemia. One of the severe complications among diabetic patients is diabetic foot ulcer (DFU), a painful inflamed ulcer accompanied by smell and pus formation, which occurs in the lower extremities, mainly the foot, and causes impairment in the functionality of tissues, bones, and joints surrounding the affected area [25,26]. Prolonged wound healing, loss of sensation, and infections are the main causes of DFU, which, if untreated, can lead to amputation. Generally, a common treatment for DFU is by administrating antibiotics to the patients. The antibiotic can be administered alone or by combining with other drugs to enhance the effectiveness to inhibit microbial growth. However, the challenging part of using antibiotics is the difficulty in eradicating the microbes due to the polymicrobial infections caused by *S. aureus*, *S. epidermidis*, *Streptococcus* sp., and *E. coli*, which are commonly found in diabetic wounds, and the continual usage of the drugs make them inefficient to eliminate the microbes or the microbes become resistant towards the treatment [27]. AgNPs are not new in biomedical applications. In the past decade, many researchers have used AgNPs in various therapeutic applications, mainly to control microbial infections in treating human biological samples. Owing to its natural antimicrobial properties, silver has been widely used in food storage, environmental applications, textile industry, and many more. With the rapid development of nanotechnology, the demand for the application of AgNPs, especially in medical settings, also increased. Indeed, wound dressings and medical equipment use AgNPs as their main vehicle to eradicate infections or inactivate bacterial growth, including multi-drug-resistant bacteria. Due to their excellent features, like nanometre in size, shape, and high surface area per volume, where a large proportion of atoms provide a better contact with the microorganisms, AgNPs can perforate into the cell walls and release the Ag + ions to kill the intracellular structures of the bacteria, thus eliminating the infections [28]. Besides AgNPs, another member of carbon-based nanoparticles, which is known as graphene quantum dots (GQDs), have also been found to be of low toxicity to cells and are widely applicable in drug delivery systems, biodetection system, and as markers for bioimaging and cellular imaging. Additionally, GQDs do present some antibacterial effects against different bacterial strains, included *S. aureus* and *E. coli*, as well by combining with other composites [29,30,31]. GQDs break down the bacterial cell wall through the activation of reactive oxygen species (ROS) and oxidative stress [32].

Basically, a wound dressing with a good basis plays a crucial role in wound regeneration. To expedite the healing stages, it should possess some properties such as the ability to promote a moist environment, accelerate wound healing, being capable to absorb excess fluid, enhance cell migration and oxygen diffusion into the pores, and most importantly inhibit infections, which is quite crucial for diabetic patients [33]. Taking all this into consideration, this study aims to fabricate a bilayer scaffold made of gauze Na-CMC as the outmost layer, into which will be incorporated nanoparticles (AgNPs/GQDs) serving as an antibacterial agent, while the collagen sponge at the bottom contains HEKs and HDFs as the epidermis and dermis layers, respectively. In this paper, GNP is chosen as a crosslinker for the bilayer scaffolds. Our goal is to characterise the chemical, physical, and mechanical properties of bilayer scaffolds and to determine the efficacy of the scaffolds using in vitro tests. Next, cell viability tests are conducted for each group and antibacterial tests for different concentrations of AgNPs/GQDs. In this manuscript, we only include the current results of the fabricated bilayer scaffolds without coated nanoparticles. The obtained results are compared to single-layer collagen, which has already been established by and characterised in our laboratory. Therefore, by adding the gauze, we want to evaluate whether the extra modification of the bilayer scaffold has similar properties to collagen or it can enhance its physicochemical properties.

## 2. Materials and Methods

### 2.1. Collagen Extraction and Stock Preparation

Prior to this study, ethical approval was obtained from the Research and Ethical Committee of the Faculty of Medicine, Universiti Kebangsaan Malaysia (Code No: UKM PP1/111/8/JEP-2020-152). The entire collagen extraction and purification processes were conducted based on the study performed by Fauzi and colleagues, with some amendments to the protocol [34]. Ovine tendon collagen type 1 (OTC-1) was used in this study. After receiving the sheep’s leg, the fur, skin, fascia, muscle tissue, and blood vessels were removed, and the tendon was cleaned. The tendon was cut and immersed in 0.35 M sterile acetic acid (Merck, Darmstadt, Germany) and stored in the chiller at 4 °C for 48 h to dissolve the collagen. After blending with acetic acid, the clear solution of collagen was coalesced with sodium chloride (0.05 g/mL) and chilled overnight at 4 °C, before being centrifuged at 5000 rpm for 5 min. Two distinct layers were formed; the upper clear solution was discarded and the turbid pellet containing collagen was immersed in cold distilled water using a dialysis tube (molecular weight cut off = 14 kDa) (Sigma-Aldrich St. Louis, MO, USA) for 72 h. The cold distilled water was changed every 12 h. The collagen solution was pre-frozen for 3 days before adding the appropriate amount of 0.35 M (pH 2.61) acetic acid for further usage. 

### 2.2. Na-CMC Gauze Preparation

The Na-CMC gauze was obtained from Cellulose Lab, Universiti Kebangsaan Malaysia (UKM), Bandar Baru Bangi, Selangor, Malaysia based on the method by Khairunnisa and colleagues, with some modifications [6]. In brief, Na-CMC powder (CAT.NO 419273) (Sigma-Aldrich, St. Louis, MO, USA) was liquefied in distilled water by using a magnetic stirrer to form a homogenous mixture. Next, the gauze (1 mm of thickness) was coated with 2% (*w*/*v*) of Na-CMC by using a wet automatic film applicator. The coated gauze was immersed in 0.5% citric acid (CA) (anhydrous, MW;192.12) (CAS NO 77-92-9) (Merck, Darmstadt, Germany) overnight. Then, the wet Na-CMC gauze was dried in an oven at 40 °C for 24 h prior to use.

### 2.3. Fabrication of Collagen Scaffold and Bilayer

**Figure 1:**
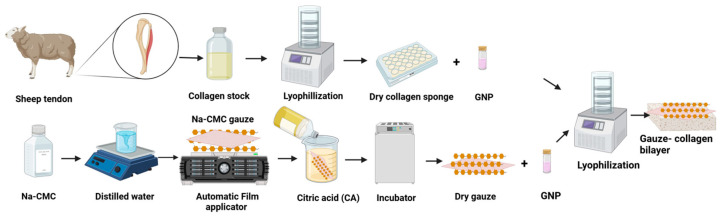
Fabrication of the bilayer scaffold (gauze–collagen bilayer).

The collagen scaffold was fabricated by pouring 2 mL of liquid collagen into the desired mould and pre-frozen at −80 °C for 6 h, before being transferred to the freeze-dryer for 48 h. For the bilayer scaffolds, 2 mL of liquid collagen was cast into a suitable mould and (1.2 × 1.2 cm) of Na-CMC gauze was cut and overlayed onto the bilayer; then, it was pre-frozen at −80 °C for 6 h, followed by freeze-drying for 36–48 h (Figure 1).

### 2.4. Fourier Transform Infrared Spectroscopy (FTIR)

The infrared spectrum produced by each scaffold was analysed by using a Perkin Elmer Spectrum 400 ATR FT-IR/NIR spectrometer (PE, Waltham, MA, USA) at the spectral range between 600 and 4000 cm^−1^ at a resolution of 2 cm^−1^ per point at room temperature.

### 2.5. Thermogravimetric Analysis (TGA)

The test was performed in a simultaneous thermal analyser (Netzsch STA 449 F3 Jupiter, Wittelsbacherstraße 42, Selb, Germany) and conducted in a liquid nitrogen environment with a heating rate of 20 °C/min, ranging from 25 °C to 600 °C.

### 2.6. Energy Dispersive X-ray Spectroscopy (EDX)

EDX was conducted to characterise the elemental compositions of the various constituents that were present in the scaffolds. The analysis was measured by using a SEM-EDX microscope (Phenom, Eindhoven, The Netherlands). Based on the critical point drying method, the scaffolds were fixed in 2% glutaraldehyde. Then, the scaffolds were washed in PBS (pH 7.4), followed by serial dilution starting at 30%, 50%, 70%, 80%, and 90% for 10 min each and 100% for 3 min. Next, the scaffolds were kept in a critical point dryer (auto-run) for 1 h and 30 min. The scaffolds were cut to the dimensions of 1 cm × 1 cm before being mounted by using carbon tape on the aluminium stub. The scaffolds were sputter-coated with gold before being viewed under a field emission scanning electron microscope (FESEM) (Supra55VP, ZEISS, Jena, Germany).

### 2.7. Porosity

The microstructural characterisation of the scaffolds was evaluated based on the porosity. The test was conducted based on the liquid displacement method by using ethanol (ChemPur, Karlsruhe, Germany) (CAS NO 64-17-5; M: 46.07 g/mol). Briefly, the volume of the scaffolds was calculated, and the initial weight was recorded. In a well plate, pure ethanol was added till the scaffolds were submerged in it. The scaffold was immersed in ethanol for 24 h at room temperature. The ethanol was removed and the excess ethanol from the scaffolds were blotted using a tissue paper, followed by weighing to obtain the final weight [9]. The porosity of the scaffolds was calculated based on the formula below:

Porosity = (W_f_ − W_i_)/ pV × 100

where W_i_ and W_f_ are the initial weight and final weight, respectively; p is the density of ethanol; and V is the volume of the scaffold.

### 2.8. Scanning Electron Microscopy (SEM) of Pore Size 

The surface and cross-section of the scaffolds were observed by using a field emission scanning electron microscope (FESEM) (Supra55VP, ZEISS, Jena, Germany). The scaffolds were cut into 1 cm × 1 cm and mounted by using carbon tape on the aluminium stub. The scaffolds were sputter-coated with gold before being viewed under FESEM. The pore sizes of the scaffolds were measured randomly by using the ImageJ software (Version 1.53, National Institutes of Health, Bethesda, MD, USA).

### 2.9. Degree of Crosslinking by Using the Ninhydrin Assay

Prior to crosslinking, the scaffolds were immersed in 0.1% (*w*/*v*) of a GNP (Fujifilm Wako Pure Chemical Corporation, Shibukawa, Japan) solution for 6 h. The GNP was discarded, and the scaffolds were washed with phosphate-buffered saline three times before pre-freezing for 6 h and freeze-dried for 3 days. The Na-CMC gauze was also crosslinked with GNP using a similar method. Furthermore, the degree of crosslinking for the fabricated scaffolds and Na-CMC gauze was determined by Ninhydrin Assay. The principle of this assay is to evaluate the percentage of free amino groups that are present in the crosslinked samples based on the absorbance readings that are comparable to the non-crosslinked samples. After GNP reacts with the amino group of collagens, the free amine group becomes less detectable by the ninhydrin reagent (2% solution, Sigma-Aldrich, St. Louis, MO, USA) [35]. For the degree of crosslinking, all the scaffolds were weighed to 10 mg and added into Eppendorf tubes containing the ninhydrin solution. The tubes were boiled for 2 min at 100 °C by using a heat blocker. After added 95% of ethanol, the solutions were transferred into 96-well plates and the amount of free amino groups was determined using optical absorbance at 570 nm with a spectrophotometer ((BioTek, Winooski, VT, USA, PowerWave) XS, Highland Park, IL, USA). For the standard, 1 mg/mL of glycine (CAS NO 56-40-6, Sigma-Aldrich, St. Louis, MO, USA) was prepared by adding 10 mg of glycine into 10 mL of distilled water. Then, the solution was mixed, and 5 different concentrations of glycine (1 mg/mL, 0.5 mg/mL, 0.25 mg/mL, 0.125 mg/mL, and 0.0625 mg/mL) were prepared. In new tubes, 200 μL of 10× ninhydrin solution and 200 μL of glycine with different concentrations were added. The tubes were boiled for 2 min, and once the solutions were cooled down at room temperature, 200 μL of 95% ethanol was added into each tube. After being resuspended, 100 μL of the solutions were transferred into 96-well plates before the absorbance reading was taken at 570 nm. The known glycine concentrations were used to plot a standard curve graph, in which the amount of free amino groups was proportional to the absorbance readings. Hence, the degree of crosslinking was calculated based on the formula below:

Degree of crosslinking (%) = (Amino_0_ − Amino_c_)/Amino_0_ × 100

where Amino_0_ is the free NH_2_ concentration in non-crosslinked groups, while Amino_c_ is the free NH_2_ concentration in the crosslinked groups.

### 2.10. Water Absorption Ability

The scaffolds were weighed (Wd) and immersed in phosphate-buffered saline (PBS) for 24 h at room temperature. After 24 h, the PBS solution was removed and the excess buffer on the scaffolds was blotted with a piece of tissue paper before the final weight (W_w_) was measured. The water absorption ability was calculated based on the formula below:

Water absorption ability (%) = (W_w_ − W_d_)/W_d_ × 100

where W_w_ is the swollen weight (g) and W_d_ is the initial weight of the scaffold (g).

### 2.11. Water Vapour Transmission Rate (WVTR)

The water vapour was measured based on the American Society for Testing and Materials (ASTM) standard [36]. Prior to the WVTR analysis, a dry scaffold was mounted on the mouth of a cylindrical glass vial that contained 10 mL of distilled water. The glass vial with the bilayer scaffold was weighed. The glass vial was then placed in an incubator at 37 °C, 5% CO_2_, and 84% relative humidity. The rate of WVTR was determined based on the formula below [37]:

WVTR = (W_i_ − W_f_)/ (A × time)

where W_i_ is the initial weight (g), W_f_ is the final weight of the scaffold (g), and A is the surface area of the cylindrical bottle.

### 2.12. Contact Angle

A total of 10 μL of distilled water was dropped onto the surface of a scaffold. By using a digital camera, the image of the water droplet on the scaffold was captured and recorded. The image was transferred, and the contact angle was measured by using ImageJ software (Version 1.53, National Institutes of Health, Bethesda, MD, USA). The wettability of the scaffold was determined based on the percentage of hydrophobicity or hydrophilicity of the scaffold. A scaffold is considered hydrophobic if the contact angle is above 90° and hydrophilic if below 90° [38].

### 2.13. Compression and Resilience

A scaffold was placed on a flat surface. By using a digital camera, the image of the scaffold was captured before compression. A load (3 N) was placed on the scaffold for 5 min. Then, the load was removed, and the image of the scaffold was captured after compression. Next, the scaffold was placed in a small beaker containing 10 mL of distilled water for 5 min. The scaffold was taken out and the image was captured after rehydration. These steps were repeated for all the other groups. The ImageJ software (Version 1.53, National Institutes of Health, Bethesda, MD, USA) was used to calculate the area of compression and resilience as per the formula below [35]:

Compression (C %) = (A_i_ − A_c_)/A_i_ × 100

where A_i_ is the area of thickness before compression and A_c_ is the area of thickness after compression.

Resilience (R %) = (A_f_/A_c_) × 100

where A_f_ is the area of thickness after rehydration and A_c_ is the area of thickness after compression. 

### 2.14. Biodegradation Rate

The 0.6% (*w*/*v*) collagenase type I powder (Worthington, Lakewood, NJ, USA) was prepared by diluting 0.6 g of the powder into 100 mL of PBS to produce 0.0006% (*w*/*v*) of the collagenase working solution. The biodegradation rate was determined through the incubation time of each scaffold in 0.0006% of collagenase at 37 °C. After incubation time at a certain period, the collagenase was removed, and the scaffolds were washed with distilled water and pre-frozen at −80 °C. The scaffolds were then subjected to freeze-drying, followed by measuring the final weight. The biodegradation rate of the scaffold was calculated based on the formula below:

Biodegradation rate (%) = (W_0_ − W_f_)/ Time

where W_0_ is the initial weight (g) and W_f_ is the final weight (g) of the scaffold.

### 2.15. Mechanical Strength

Scaffolds were prepared in a 1 cm × 3 cm size. Both edges of the bilayer scaffolds were secured with masking tape. The thicknesses of the two ends as well as of the middle of each scaffold were measured in millimetres (mm) by using a vernier caliper. The mechanical strength of the scaffold was evaluated by using the Instron Universal Testing Machine model 5557 (Instron, Norwood, MA, USA). The machine had a 500 N load cell and mechanical force at the rate of 5 mm/min, which was integrated with the Instron Bluehill^®^ 3 Software. The scaffolds were inserted one at a time between the clamps (10 mm in diameter and 1 mm in height) to avoid slipping during testing. Triplicate scaffolds from each group were tested to obtain the average value for ultimate tensile strength, Young’s modulus, and elongation at break. All the tests were conducted at room temperature. The tensile strength was evaluated based on the formula below [39]:

*E* = *σ*/*ε*

where σ is the Young’s modulus (compressive force per unit area or stress) and ε is the changes in volume per unit volume (strain).

### 2.16. Skin Cell Processing

The skin sample collection was approved by the Research and Ethical Committee of the Faculty of Medicine, UKM (Code No: UKM PP1/111/8/JEP-2020-152). Healthy skin samples were procured from patients who underwent circumcision or abdominoplasty. Once received, the skin sample was cut to the dimensions of 1 cm x 3 cm. The sample was cleaned to remove thin fat, hair, debris, blood, and flesh. After being washed in Dulbecco’s phosphate-buffered saline (DPBS), the sample was cut and minced into smaller pieces of about 1 mm^2^. The skin sample was then added to a tube containing 0.6% collagenase type I (Worthington, Lakewood, NJ, USA) and incubated in an incubator shaker at 37 °C for 4–6 h. Next, 0.05% trypsin EDTA (Gibco, Carlsbad, CA, USA) was added into the tube to dissociate the cells for 10 to 15 min in an incubator shaker. To the cells was added with F-12: Dulbecco’s Modified Eagle Medium (FD; Gibco, Carlsbad, CA, USA) to neutralise the trypsin. After being centrifuged, the cells were washed, and any undigested large tissue was discarded. The skin that was digested contained human epidermal keratinocytes (HEKs) and human dermal fibroblasts (HDFs). The cells were resuspended in a six-well culture plate, containing the medium Epilife (Gibco/BRL, Grand Island, NJ, USA) and FDC with 10% foetal bovine serum (FBS; Biowest, Riverside, MO, USA). The plate was then incubated at 37 °C in an incubator containing 5% CO_2_ and the medium was replaced every 48 h. Once the cells reached 80% confluency, to the cells was added 0.05% Trypsin-EDTA for 5 min to dissociate the adherent cells (HDFs), and they were transferred into a new flask, while HEKs remained on the plate. Both the cells in the flask and on the plate received complete medium of FD containing 10% FBS (FDC) and were incubated further to obtain the desired confluency for the cell toxicity and proliferation tests.

### 2.17. Cell Viability

#### 2.17.1. Live and Dead Assay

Live and dead assay (LIVE/DEAD Cell Viability Assay (Invitrogen, Waltham, MA, USA) was performed to determine the cytotoxicity effects and viability of the collagen and bilayer scaffolds after being seeded with HEKs and HDFs. Firstly, the scaffolds were sterilised with 70% alcohol. After the sterilisation process, the cells were seeded independently onto the scaffolds and stabilised in an incubator for 24 h. The scaffolds were stained with 2 mM Calcein AM and 4 mM ethidium homodimer-1 (EthD-1) in a pure culture medium and kept in an incubator for ½ hour. After discarding the stain, the scaffolds were washed with DPBS before being viewed under a confocal laser scanning microscope with a Nikon A1R-A1 camera (CLSM; Nikon, Tokyo, Japan).

#### 2.17.2. MTT Assay

3-(4,5-dimethylthiazol-2-yl)-2,5-diphenyltetrazolium bromide (MTT) (Thermo Fisher Scientific, Waltham, MA, USA) was used to measure the cellular metabolic activity, which indicates the number of viable cells of both HEKs and HDFs on the scaffolds. Firstly, the MTT stain was prepared by mixing 5 mg of MTT reagent into 1 mL of sterile DPBS. Then, HEKs and HDFs were seeded onto the scaffolds before being incubated for 24 h. Three-well plates were used to seed both cells independently for three different time frames (Day 1, Day 3, and Day 7). After discarding the old medium from the plate, 100 μL of the complete medium (FDC) was added into each well along with 10 μL of MTT stain, wrapped in aluminium foil, and incubated at 37 °C, 5% CO_2_ for 4 h. All the stain was removed, except 25 uL in each well. Then, 75 μL of DMSO was added into each well to make a total of 100 μL. The plates were incubated further for another 10 min at room temperature before the absorbance reading was taken using a spectrophotometer at 570 nm wavelength. These steps were repeated for Day 3 and Day 7.

### 2.18. Minimum Inhibitory Concentration (MIC)

The minimum inhibitory concentration test was performed based on the protocol by Wei et al., with some modifications [40]. The AgNPs were obtained from our collaborator, the Faculty of Engineering and Built Environment, UKM, Bangi and GQDs (Blue Luminescent) from ACS Material, Pasadena, CA, USA. Initially, 100 μL of *S. aureus* and *E. coli* suspensions from an overnight culture were added into two separate tubes containing 10 mL of Mueller–Hinton (Sigma-Aldrich, Burlington, MA, USA) broth. Different concentrations of AgNPs and GQDs were prepared (1 mg/mL, 0.5 mg/mL, 0.25 mg/mL, 0.125 mg/mL, and 0.0625 mg/mL), and 100 μL of each concentration was added into wells 1–5 in two 96-well plates. The technique for serial concentrations of AgNPs was adapted based on Atiqah et al. and Erenler et al., with some changes after optimisation. To compare the bacterial effects, concentrations of GQDs were prepared using a similar method. The same steps were repeated for gentamicin (positive control) [9,41]. Then, 100 μL of bacterial broth was also added to all the wells. Broth only without bacteria represents the negative control. The plates were incubated at 37 °C overnight. The absorbance reading was recorded at 600 nm by using a microplate reader (Multiscan GO, Thermo Scientific, Ratastie 2, Vantaa, Finland).

### 2.19. Statistical Analysis

The data were analysed by using GraphPad Prism version 8.0, San Diego, CA, USA. The data collected are presented as the mean ± SD (standard deviation) of the scaffold size. A one-way ANOVA analysis was used for the comparison of one group with the other groups. The results are presented as mean and standard deviation and a *p*-value < 0.05 was considered as a significant value. The legends in the following graphs represent the scaffolds: NCG (non-crosslinked gauze with GNP), CLG (crosslinked gauze with GNP), NCC (non-crosslinked collagen with GNP), CLC (crosslinked collagen with GNP), NCB (non-crosslinked bilayer with GNP), and CLB (crosslinked bilayer with GNP).

## 3. Results

### 3.1. Gross Appearance

**Figure 2:**
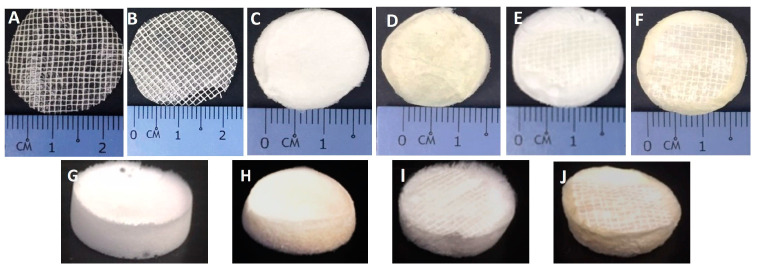
Gross appearance of the different scaffolds. (**A**) NCG. (**B**) CLG. (**C**) NCC. (**D**) CLC. (**E**) NCB. (**F**) CLB. (**G**) NCC scaffold. (**H**) CLC scaffold. (**I**) NCB bilayer scaffold. (**J**) CLB bilayer scaffold.

Figure 2 demonstrates the gross appearance (surface layer and 3D structure) of different scaffold groups before and after crosslinked with GNP. It shows that image A and B are gauze alone containing Na-CMC; C and D are collagen scaffolds; and E and F are bilayer scaffolds (collagen and Na-CMC gauze). Images G to J represent 3D structure of the scaffolds, with G and H are collagens and I and J are bilayers. After being crosslinked with GNP, the NC and CL groups exhibited a non-transparent (opaque) outer layer in structure. NCG, NCC, and NCB remained milky white in colour, while CLG, CLC, and CLB appeared to be light brownish in colour. In previous papers, authors have reported that, upon reacting with GNP, the material turns blue. The colour changes from yellowish to dark blue are merely due to the duration of crosslinking between GNP and the scaffolds [42,43]. 

### 3.2. Chemical Characterisation of Bilayer Scaffolds (FTIR, TGA, and EDX)

#### 3.2.1. Fourier Transform Infrared (FTIR)

**Figure 3:**
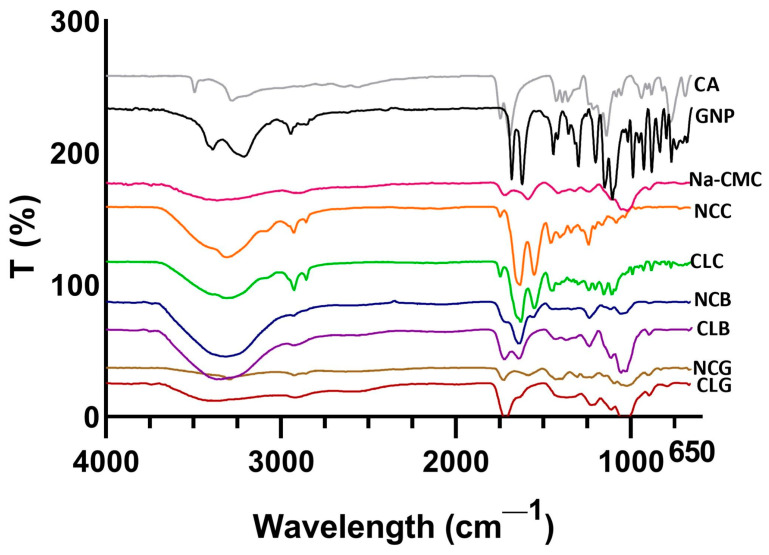
FTIR spectrum analysis between non-crosslinked and crosslinked groups.

The FTIR spectra of the CA, GNP, Na-CMC, collagen scaffolds, bilayer scaffolds, and gauze are shown in Figure 3. The CA spectrum was visible at 3279 cm^−1^ caused by the O-H stretching of water molecules, while the C=O stretching of carboxylic acids caused peaks at 1743 cm^−1^ and 1692 cm^−1^. The obtained peaks for NLG after crosslinking with CA were at 3293 cm^−1^ and 1726 cm^−1^ and CLG was situated at the peak at 1714 cm^−1^ (C=O stretching); the peak at 1111 cm^−1^ was due to the C-OH stretching vibration. For CLB, peaks were observed at 1721 cm^−1^, 1638 cm^−1^, and 1112 cm^−1^, while for NCB, at 1637 cm^−1^ and 1117 cm^−1^ [6,44]. Overall, the bands at 1721–1741 cm^−1^ were related to CMC-CA [6]. However, except for NCG, major bands (3293 cm^−1^) were not visible for the scaffolds, probably due to the crosslinking with GNP. The spectrum of GNP demonstrated an absorption peak observed at 1679 cm^−1^, assigned to the stretching vibrations of C=C of the carboxymethyl group, and another absorption peak at 1618 cm^−1^ was attributed to the C=C vibration of GNP. The C-H stretching vibration caused a peak to appear at 2800–3000 cm^− 1^. The double peak between 3000 and 3400 cm^− 1^ in the GNP spectrum is most probably due to the overlapping of the aromatic C–H and O–H vibration bands [45]. The FTIR spectra of Na-CMC showed broad peaks, observed at three peaks. The peak at 1585 cm^−1^ was caused by the COO^−^ stretching vibrations of the carbonyl group; the peak at 1413 cm^−1^ was due to CH_2_ scissoring, which is a carboxymethyl group as it is salt; and the third peak at 1319 cm^−1^ was assigned to the OH bending vibrations of Na-CMC. Moreover, the distinctive peaks of Na-CMC were observed between 1237 cm^−1^ and 1019 cm^−1^. In a similar study by Dharmalingam and the team., a peak was noticeable at 893 cm^−1^, which was assigned to C-O-C stretching vibrations, referring to the Na-CMC peak as well [46]. In the bilayer scaffolds and gauzes, absorbable peaks were observed between 2920 cm^−1^ and 2922 cm^−1^, which were attributed to the N-H stretching of Na-CMC [47], and as explained earlier, the peaks at 1580–1590 cm^−1^ were due to the COO^−^ stretching vibrations of carbonyl groups and the bands at 1416–1422 cm^−1^ were assigned to CH_2_ scissoring [47,48]. 

The observed spectrum of OTC-1 for all collagen and bilayer groups showed peaks of N-H stretching at 3311–3309 cm^−1^ for Amide A. The CH_2_ symmetrical stretching at 2925–2923 cm^−1^ corresponds to Amide B, Amide I at 1632–1627 cm^−1^, Amide II due to C-N stretching vibrations at 1550–1549 cm^−1^, and Amide III at 1239–1298 cm^−1^ [34,49]. Despite this, the collagen scaffolds peaks were in the range between 1454 cm^−1^ and 1239 cm^−1^, which correspond to the collagen helical structure, while the peak at 1632 cm^−1^ indicates the beta-sheet or triple-helix structures of collagen. The percentage of crosslinking between GNP and CLG is quite low, which explains the lower detection of GNP in the gauze [49].

#### 3.2.2. Thermogravimetric Analysis (TGA)

**Figure 4:**
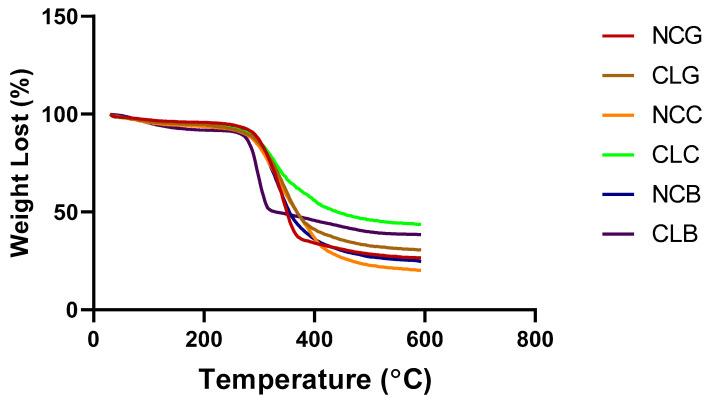
TGA analysis between non-crosslinked and crosslinked groups.

The thermal stability of the scaffolds is depicted in Figure 4 and the degradation steps of weight loss is presented in Table 1. In the present study, the TGA analysis showed a weight reduction in all the scaffolds during the heating process between 0 °C and 600 °C. There were four stages involved in weight reduction during the process starting from 0 °C to 600 °C. In the first phase at the lowest temperature (30 °C), there were no differences in the percentage of weight loss for all the groups. In the second stage, vaporisation and a weight reduction of 10% occurred at the temperature of 180–300 °C for all scaffolds, except for CLB, which showed a 30% of weight loss. The weight reduction in the third stage occurred at a temperature of 350 °C–400 °C, where the water loss was about 60% in all the NC groups and 50% in the CL groups. In the fourth stage, a temperature ranging from 490 °C to 600 °C caused a weight loss of around 70% in all NC groups and a 60% reduction was observed for the CL groups [50]. The weight loss % in the NCB scaffolds was relatively higher than that of the CLB scaffolds, while NCC showed the highest reduction in weight of all. CLC exhibited the lowest reduction in weight (43.65%), followed by CLB (38.45%), CLG (30.58%), NCG (26.2%), NCB (24.65%), and NCC (20.13%) at the temperature of 593 °C.

#### 3.2.3. Energy Dispersive X-ray Spectrometry (EDX)

Table 2 categorised the elemental compositions and distributions of each group based on the percentage by using EDX spectroscopy. EDX detects the homogeneity of the scaffolds, where carbon, oxygen, and nitrogen are the main elements in collagen scaffolds while carbon, oxygen, and natrium in gauze and bilayer scaffolds. Comparing each pair of CL and NC scaffolds, carbon elements were highly detected in NC groups. Following this, NCG showed a percentage of carbon at 40.59 ± 0.82%, followed by NCC (58.04 ± 0.09%) and NCB (41.40 ± 0.83%), whereas in CLG, the percentage of C was around 37.80 ± 0.67%, 48.50 ± 0.23% for CLC and 36.10 ± 0.43% for CLB. Meanwhile, there was not much difference in O elements in NCG, CLG, NCB, and CLB, and the presence of Na was detected in these scaffolds, indicating salt from Na-CMC.

#### 3.2.4. Porosity, Pore Size, and SEM

**Figure 5:**
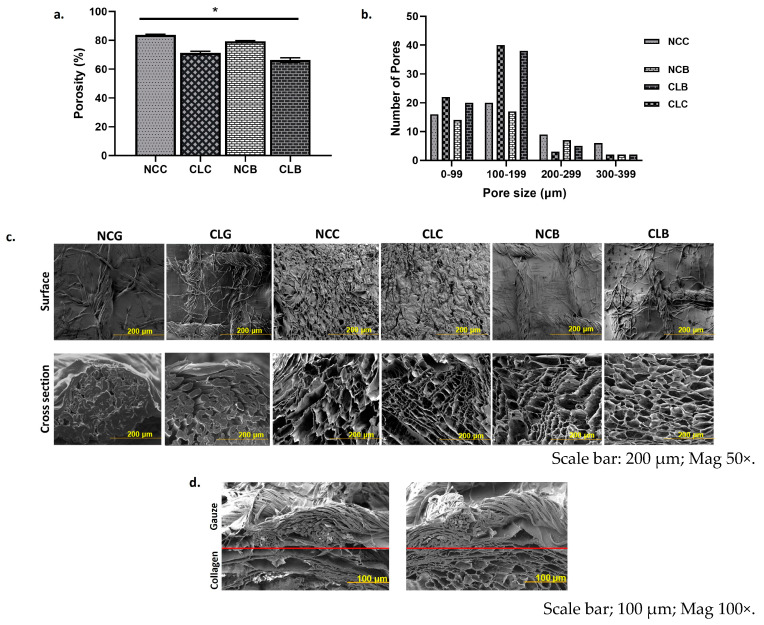
The morphological structures of the bilayer scaffolds. (**a**) Percentage of porosity. (**b**) Number of pores and SEM morphology of the bilayer scaffolds. (**c**) Gross appearance. Surface and cross-section of the bilayer scaffolds. (**d**) Bilayer scaffolds of non-crosslinked and crosslinked groups. Red lines denote the border line between gauze and collagen. (*) represents a significant difference (*p* < 0.05) between non-crosslinked and crosslinked groups.

Figure 5a represents the percentage of porosity for non-crosslinked and crosslinked scaffolds. From the graph, we can observe that both groups exhibited a porosity of more than 50%. NCC and NCB had the highest percentage of porosity than the CL groups with the value of 83.67 ± 0.58% and 79.33 ± 0.58%, respectively. On the other hand, the percentage of porosity for CLC was 71.33 ± 1.16 and for CLB 66.33 ± 1.53. The reduction in the percentage of porosity was due to the crosslink of 0.1% of GNP with the amine group of the collagen. This result tallies with the result in Figure 5b, where the pore size of the collagen and bilayer scaffolds gradually decreased after crosslinking with GNP. NCC showed the highest pore size of 200–399 μm, followed by NCB in the range of 200–299 μm. Both CLC and CLB had the highest pore size of 100–199 μm. Similarly, the SEM result in Figure 5c for the CL scaffolds shows a good distribution of the porous microstructure, which corresponds to a suitable porosity and pore size. Figure 5d shows SEM of bilayer scaffolds (top gauze and bottom collagen) for NCB and CLB. 

#### 3.2.5. Degree of Crosslinking

**Figure 6:**
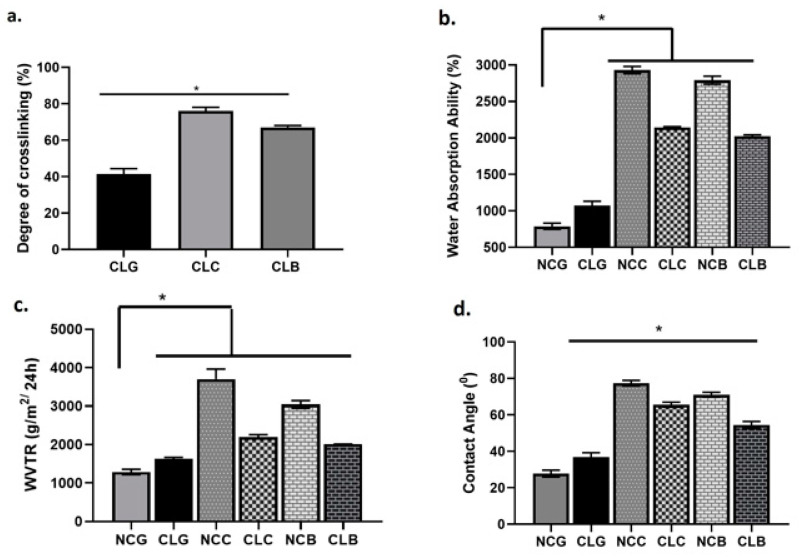
(**a**) Degree of crosslinking. (**b**) Water absorption ability. (**c**) Water vapour transmission rate. (**d**) Contact angle. (*) represents a significant difference (*p* < 0.05) between NC and CL groups.

Figure 6a shows the degree of crosslinking in percentage for the CLG, CLC, and CLB groups. Among the crosslinked groups, CLC demonstrated the highest percentage with 76.00 ± 2.00%, followed CLB with 67.33 ± 1.00% and CLG with 41.33 ± 1.76%. The degree of crosslinking refers to the amount of the crosslinking GNPs that reacted with the amine groups of the collagen and bind to each other. This was confirmed after adding the ninhydrin reagent, which was used to assess the presence of amino acids and proteins in the free NH_2_ group. Therefore, the higher the percentage of the crosslinking, the lower the amount of free amine groups in the CL groups [51]. 

#### 3.2.6. Water Absorption Ability, Water Vapour Transmission Rate, and Contact Angle

Figure 6b shows the water absorption ability of the scaffolds. All the scaffolds showed a water absorption ability of more than 1000%, except for NCG, which was 789 ± 42.28% and CLG at 1074 ± 59.27, while CLC and CLB had a water absorption ability with values of 2142 ± 13.27% and 1993 ± 17.37%, respectively. However, NCC demonstrated the highest water absorption ability, at 2930 ± 50.93%, compared to NCB at 2792 ± 55.42%. Except for the gauze groups, the non-crosslinked scaffolds exhibited higher levels of water absorption, as these scaffolds were not crosslinked with GNP and collagen had a high capability to absorb water molecules. 

The water vapour transmission rate (WVTR) is shown in Figure 6c. The purpose of this test was to evaluate the moisture permeability of the scaffolds. It is shown that CLC, CLB, and CLG exhibited WVTRs with mean values of 2194 ± 66.81 gm^2^/24 h, 2013 ± 44.09 gm^2^/24 h, and 1460 ± 53.49 gm^2^/24 h, while NCG, NCC, NCB, and NCC had WVTRs with values of 3701 ± 264.7 gm^2^/24 h, 3043 ± 100.2 gm^2^/24 h, and 1189 ± 71.31 gm^2^/24 h, respectively. The WVTR for the non-crosslinked groups was higher than that of the crosslinked groups. These results show that the higher the percentage of the swelling ratio, the higher the WVTR. 

The contact angle of the non-crosslinked and crosslinked groups is shown in Figure 6d. Both groups exhibited a contact angle below 90°, which is hydrophilic. From the graph, the degree of the contact angle for CLC is 65.58 ± 1.35° and for CLB is 54.48 ± 1.90°, while CLG exhibited a contact angle at 36.80 ± 2.39°. Meanwhile, NCG, NCC, and NCB exhibited contact angles with the values of 27.73 ± 1.89°, 77.31 ± 1.57°, and 71.05 ± 1.35°, respectively. Even though NCC and NCB showed the highest contact angle compared to the other scaffolds, all the scaffolds showed a good surface wettability.

### 3.3. Mechanical Properties of Bilayer Scaffolds

#### 3.3.1. Compression, Resilience, and Biodegradation

**Figure 7:**
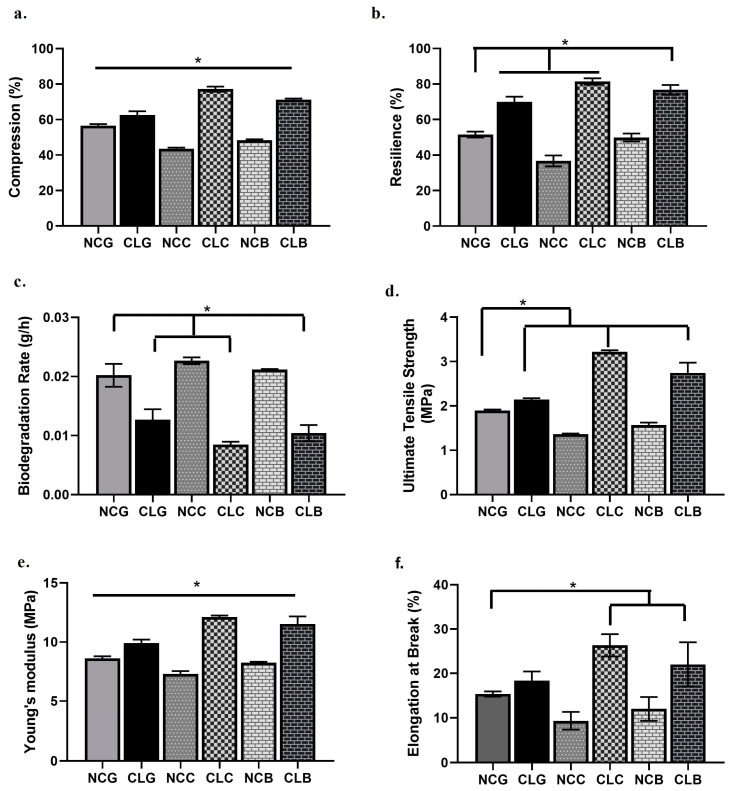
(**a**) Compression. (**b**) Resilience. (**c**) Biodegradation. (**d**) Ultimate tensile strength. (**e**) Young’s modulus. (**f**) Elongation at break. (*) represents a significant difference (*p* < 0.05) between non-crosslinked and crosslinked groups.

In Figure 7a, all CL groups show a lower percentage of compression than the non-crosslinked groups. Among the samples, CLC had the highest compression, which was 77.18 ± 1.45%, while CLB and CLG had 71.22 ± 0.69% and 62.54 ± 2.20%, respectively. For the non-crosslinked samples, NCC had the highest percentage of compression, which was 56.55 ± 0.93%, followed by NCB (48.33 ± 0.58%) and NCG (43.51 ± 0.70%). For the scaffold that returned to their original shape and size after being immersed in water (Figure 7b), CLC had the highest percentage of resilience, with a value of 81.40 ± 1.82%. CLB had the second highest percentage of resilience, with a value of 75.03 ± 1.34%, followed by CLG with 70.03 ± 2.88%. All the non-crosslinked scaffolds exhibited a lower percentage of resilience: NCG (51.56 ± 1.68%), NCB (49.85 ± 2.24%), and NCC (36.71 ± 3.06%). Figure 7c shows the reduction in mass in scaffolds. The results display that the scaffolds after being crosslinked with GNP have the strongest resistance towards degradation than the scaffolds without GNP. CLC had the lowest degradation rate at 0.008 ± 0.001 g/h, comparable to CLB (0.010 ± 0.002 g/h) and CLG (0.013 ± 0.002 g/h). There was no significant difference in the degradation rate observed between CLC and CLB. This can be due to GNP, which could have delayed the degradation process and improved the stability of the prepared scaffolds, especially CLB, which is applicable for wound healing. For the non-crosslinked groups, NCC had the highest degradation rate, with 0.0227 ± 0.0006 g/h, followed by NCB at 0.02116 ± 0.0001 g/h and NCG at 0.0202 ± 0.002 g/h. 

#### 3.3.2. Ultimate Tensile Strength, Young’s Modulus, Elongation at Break

Other mechanical properties of non-crosslinked and crosslinked scaffolds were evaluated based on three tests, namely ultimate tensile strength, Young’s modulus, and elongation at break (Figure 7). The principle of ultimate tensile strength is based on the maximum stress that a scaffold can withstand while being stretched or pulled before it breaks [52]. In the graph in Figure 7d, the ultimate tensile strength increased in CLC (3.22 ± 0.04 MPa), CLB (2.74 ± 0.23 MPa), and CLG (2.14 ± 0.04 MPa) and decreased in NCG (1.89 ± 0.03 MPa), NCB (1.57 ± 0.06 MPa), and NCC (1.37 ± 0.02 MPa). Here, it is revealed that the GNP 0.1% crosslinked scaffolds were able to resist more stress applied on the scaffolds. Even though CLC had the highest MPa value for ultimate tensile strength, CLB also showed a high MPa value with the addition of CMC-CA crosslinking to the gauze. Young’s modulus or elastic modulus is related to the stiffness of a material, whether the material can be easily bent or stretched. In Figure 7e, the Young’s modulus values for the crosslinked scaffolds were 12.11 ± 0.12 MPa, 11.52 ± 0.66 MPa, and 9.91 ± 0.30 MPa with respect to CLC, CLB, and CLG, while 8.62 ± 0.17 MPa, 8.25 ± 0.08 MPa, and 7.29 ± 0.25 MPa corresponded to NCG, NCB, and NCC, respectively. The crosslinked bilayer scaffolds had a higher modulus than the non-crosslinked scaffolds. The elongation at break is the percentage increase in length that a scaffold will achieve before breaking. The highest elongation at break (%) values for the crosslinked scaffolds, as shown in Figure 7f, were for CLC (26.33 ± 2.52%), CLB (22 ± 5.00%), and CLG (18.33 ± 2.08%), while the lowest elongation at break values were for NCG (15.33 ± 0.58%), NCB (12.00 ± 2.65%), and NCC (9.33 ± 1.94%).

### 3.4. Cell Viability Analysis (Live and Dead Assay and MTT Assay) 

**Figure 8:**
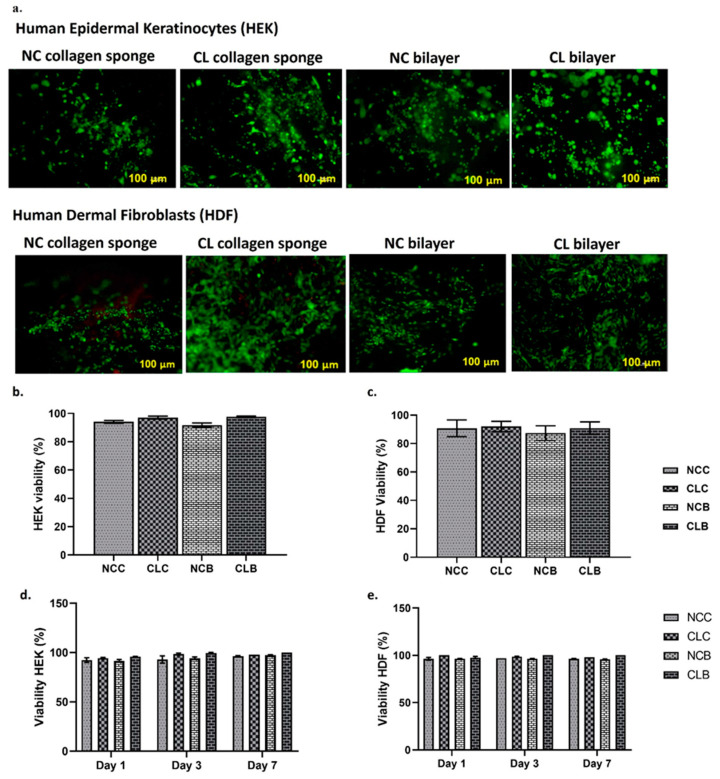
Live/dead cell viability assay. (**a**) The qualitative analysis of cell viability by using HEKs and HDF cells on bilayer scaffolds. (**b**,**c**) The quantitative analysis of HEKs and HDFs cells in 24 h of incubation based on the percentage of live/dead cells. (**d**,**e**) MTT assay based on the percentage of cell viability for HEKs and HDFs on Days 1, 3, and 7.

Figure 8a shows the fluorescence images of the live (green)/dead (red) cells on the collagen and bilayer scaffolds after 24 h of cultivation. Here, we did not include the data for CLG and NCG because, due to the porous structure, no cell was detected after staining. According to the cell viability tests (qualitative), both crosslinked and non-crosslinked groups of the collagen and bilayers had a good cell viability, which can be seen in the images. Based on Fauzi et al. (2017), OTC-1 was expected to have no dead cells, which agrees with our findings [34]. In addition, it can be observed that the collagen and bilayer groups contained viable cells (green stain) with negligible dead cells (red stain), further suggesting that HEKs and HDFs grew well on these scaffolds during the incubation time. In Figure 8b,c, the quantitative analysis of HEKs and HDFs cells showed a cell viability greater than 90% in the collagen and bilayer scaffolds. Meanwhile, MTT test was utilised to further determine the cell viability of HEKs and HDFs seeded on NCC, CLC, NCB, and CLB in three different time frames (Day 1, Day 3, and Day 7). The results indicate that the scaffolds are non-toxic to human skin cells. The principle of the MTT assay is based on the changes in MTT (a yellow salt) with respect to formazan (an insoluble purple substance), with the presence of dehydrogenase enzymes contained in the mitochondria of living cells at 37 °C [53]. As presented in Figure 8d,e, there is not much significant difference in the percentage of cell viability for Days 1, 3, and 7 for both non-crosslinked and crosslinked groups. Both groups showed a cell viability of more than 90%. To be specific, the MTT results exhibit the high viability of HEKs and HDFs on Day 7 for CLC and CLB, which is 100% comparable to the NC scaffolds. These results verify the cytocompatibility of the fabricated CLB, which is similar to that of CLC. Of note, the presence of Na-CMC and CA in CLB shows that they are biocompatible materials whose combination resulted in the production of non-toxic bilayer scaffolds for HEKs and HDFs, whilst enhancing the cells’ growth and viability to hasten wound closure and reduce scar formation [4,14,54].

### 3.5. Minimum Inhibitory Concentration (MIC)

Table 3 shows the minimum inhibitory concentrations of AgNPs and GQDs against *S. aureus* and *E. coli*. The results demonstrate that AgNPs inhibit the growth of *S. aureus* and *E. coli* at the minimal concentrations of 0.25 mg/mL and 0.125 mg/mL, respectively. On the other hand, GQDs exhibited the lowest concentration at 0.5 mg/mL and gentamicin at the concentration of 0.0625 mg/mL for both bacterial strains. Based on the table below, AgNPs and GQDs possess some bacteriostatic effects against *S. aureus* and *E. coli*.

## 4. Discussion

Overall, this study demonstrated the fabrication of a versatile sponge scaffold using OTC-1 and Na-CMC gauze on the top layer for the treatment of full-thickness skin wounds. These combinations after crosslinked with GNP had a very good water absorption ability, contact angle, WVTR, porosity, and mechanical and chemical properties, which can be altered to suit the requirements of wound healing through the selection of crosslinking agents and the concentration. In this study, collagen was modified by adding another layer on top, which had Na-CMC gauze crosslinked with CA. Though the main portion of the scaffold is collagen, the difference with the layered gauze was evaluated. The use of collagen as a sponge can mimic the extracellular matrix of the human body, and the gauze with CA after being coated with nanoparticles serves as an antibacterial barrier that can be applied in future studies of diabetic wounds.

Overall, the bands at 1721–1741 cm^−1^ were related to CMC-CA in NCG, CLG, NCB, and CLB [6]. In the FTIR analysis, the typical spectra peaks for collagen indicated the presence of Amide I, II, and III between 1632 cm^−1^ and 1239 cm^−1^ [35]. Additionally, the absorbance peak at 1618 cm^−1^ was due to the C=C vibration of the olefin ring in GNP [45]. Amide I, II, and III after being crosslinked with GNP exhibited no distinct peak formation in the CL groups. Except for Amide III, a slight peak shift was observed for Amide I and II, a similar finding to that of Fauzi and the team [14]. The spectra peak between 1237 cm^−1^ and 1019 cm^−1^ indicated the presence of a glucopyranose ring in Na-CMC. A sugar ring was also observed in the gauze and bilayer scaffolds [46]. There was also the presence of intermolecular and intramolecular hydrogen bonds and OH group, which formed a visible broader band for cellulose that can be seen at 3293–3335 cm^−1^ for NCG, CLG, NCB, and CLB, while at 3363 cm^−1^ for Na-CMC [46]. The FTIR of each bilayer scaffold represents a spectral wavelength corresponding to the protein structure characterisation of Na-CMC, CA, and collagen based on the stretching vibrations at different regions [6,55,56]. 

For the TGA analysis, the results indicate that the CL groups were able to withstand high temperatures and retained the longevity of the scaffolds (CLC, CLB, and CLG). However, a reduction in weight still occurred in these scaffolds due to the reduced stability of the collagen and the disintegration of the carboxymethylcellulose carbonyl groups in the bilayer scaffolds and gauze [57,58]. In an exceptional case, a weight loss around 60% was observed for CLC. Probably, more low stability and volatility might have occurred for collagen scaffold at the temperature of 180–300 °C, which could explain the weight loss around 60%. However, in the final degradation step, there was not much difference in weight loss that could be seen between CLC and CLB. More weight loss was observed at a temperature range of 350–400 °C. According to Nusaibah and colleagues, it is essential to maintain the thermal stability of a scaffold to prevent any damage due to harsh conditions and to prolong the usage of the scaffold after implantation in chronic wound. They added that the non-crosslinked scaffolds with GNP have a low thermal stability in comparison to the crosslinked scaffolds because non-crosslinked groups have collagen with a triple helix chain and large enthalpy [59]. 

Furthermore, the presence of an endothermic peak in the graph, caused by the transition of the helix coil as the heat broke the hydrogen bonds of the collagen, caused the collagen to be eliminated. This explains the reduction in weight in the first stage and in the second stage that can be caused by splitting the chain of the organic components in the biomaterial. The first phase at 180–300 °C is known as the volatile phase, in which the excess water and other substances present in the scaffold are removed. Probably, more substances were removed, which could explain the higher weight reduction in CLB during this phase. A major reduction in weight for all the groups was observed between 350 and 400 °C as the volatile products were destructed or decomposed, while the polysaccharide rings lost more water through the evaporation process [59,60]. It is also reported that the CA hydrogen bond could increase the thermal stability and avoid any retrodegradation of the material. Thus, both 0.5% of CA and 0.1% of GNP crosslinkers might resist the high thermal stability that caused a lower weight lost in the CLB scaffold in this phase [60]. A previous research article reported that CMC had a high-water content based on the results of a TGA. The article further explored the TGA, and the results showed that there were still traces of water molecules after it was left to dry in a vacuum oven at 50 °C for 24 h. The presence of water molecules could be due to the hydrogen bonds that are formed between the water molecules and the polymer chains of the Na-CMC. Additionally, the substitution of the O_6_H_6_ groups with CH_2_COONa also influenced the hydrogen bond structure (Na-CMC) to increase water intake, making the bond more complex to sustain its hydrophilicity [61]. The last phase of the TGA was combustion, in which all the scaffolds reacted with the atmospheric gases like nitrogen and turned into ashes. In the EDX analysis, it was revealed that the main elements, C and O, are present in the collagen and bilayers, except for Na elements, which were found only in the gauze and the bilayer groups, which indicates the presence of salt in the gauze and the bilayer groups. The crosslinked scaffold with GNP showed a lower presence of C elements. All the elements were distributed homogeneously in the scaffolds [35]. 

A pore size of 100–200 μm is acceptable for cell migration and diffusion of oxygen and nutrients, ideal for skin wound healing [14]. NCC and NCB have a higher percentage of porosity than CLC and CLB because the NC scaffolds did not shrink in their interpore structures after crosslinking. Both CLC and CLB scaffolds have a porosity of more than >50%, which is vital to allow oxygen diffusion and the translocation of essential nutrients for cell migration, multiplication, and formation of blood vessels [14]. Based on the porosity data, CLC and CLB have suitable pore sizes of 100–199 μm, whereby most of the pores are within the ideal range. As for the SEM images, the good distribution of the porous microstructure for CLC and CLB was due to the pre-freezing and lyophilisation techniques. In this study, after being crosslinked with GNP, the collagen and bilayer scaffold were pre-frozen at −80 °C, followed by lyophilisation or freeze-drying [62]. The purpose of these techniques was to remove the solvent while retaining the scaffolds with a good porosity and controlling the pore size. It was also reported that maintaining the freezing temperature is quite vital during the manufacturing of the scaffolds as the temperature of the freezing rate rises, which has a high possibility to form ice crystals in the pores and could hinder the overall function of the new cells [63,64]. 

Naturally, collagen type 1 has a poor mechanical structure and is frail. Crosslinking with GNP improves its strength and structure and prevents degradation. Owing to its low toxicity to cells and low immunogenicity, a high concentration of GNP results in cytotoxicity to cells. Therefore, in this study, after optimisation, 0.1% GNP was chosen to crosslink with collagen [65]. The purpose of crosslinking Na-CMC with GNP in this study was to investigate the physicochemical characterisation of the gauze alone. Moreover, the recent literature has mentioned that GNP has the capability to crosslink with polysaccharide-based materials, like chitosan, and produces a good physical barrier for cellulose composites [65,66], but our findings reveal that, after crosslinking with GNP, the gauze Na-CMC (CLG) confers a lower percentage of crosslinking with GNP compared to CLB. Probably, the GNP solution would work better directly with an Na-CMC solution, which could facilitate the crosslinking mechanism before freeze-drying the material [66]. On the contrary, the bilayer had a better crosslinking due to the presence of collagen at the bottom layer, which could blend well with GNP.

Despite the advantages of using CMC in various therapeutic applications, poor mechanical strength becomes one of its major drawbacks. The CMC gauze is unable to retain its structure and collapses once it touches water [66]. In this study, prior to being crosslinked with GNP, Na-CMC (gauze) was crosslinked with CA to increase the physical–chemical integrity and permeability of the gauze. The crosslinking of CA with Na-CMC was initiated through the esterification process. This resulted in the formation of an ester bond by intermolecular crosslinking between cyclic anhydride moiety and the hydroxyl groups of cellulosic chains [46]. Additionally, the stability of the CMC structure enables it to mix with other water-dissolving polymers, such as ethylene glycol, or natural polymers, like collagen, having a high affinity towards skin, bone, and mucous membranes. CMC is used as a product for wound dressing as it can elevate the cytokine level in wounds and trigger the activation of macrophages. During chronic wound healing, the water absorption ability of CMC can support wound regeneration and ECM formation [4].

Water absorption ability is a measurement to evaluate the ability of a scaffold to absorb water, including excessive exudates, as it is important to provide a moist environment to accelerate wound healing after implantations in vivo, especially in diabetic wounds [67]. Both CLC and CLB have a lower water absorption ability than the NC groups. This could be due to the hydrophilicity of the collagen and bilayer scaffolds decreasing as the heterocyclic compound of the collagen was altered; the increased formation of new bonds reduces the swelling ratio or water absorption ability of the scaffolds [19]. In CLG, despite the presence of CA, the water uptake is still high compared to NCG. This could be due to a smaller percentage of GNP being crosslinked with CLG. However, in comparison to CLG and CLB, it is still considered lower. The logical reason for this can be the primary amine of GNP that changed after crosslinking with the polysaccharide group of the gauze [65]. In contrast, 0.5% of CA in NCG is too low to obtain a good water absorption ability [68]. A study in the literature also stated that an increase in the concentration of CA would increase the water uptake of CMC, especially when it reached up to 10% of the concentration [69]. However, CLC and CLB are within the ideal range for water absorption ability. In addition, this physical property is equally important to reduce the formation of scars while reducing pain and preventing infections from the external environment. The disadvantage of high water absorption in a scaffold could lead to an increase in pore size and porosity to a degree or extent in which the scaffold attracts more water (hydrophilic). According to Atiqah and colleagues, the ideal range for water absorption ability is more than 1000%, which is an acceptable range for skin wound closure [9]. This will help in cell adhesion and proliferation [70]. Apart from this, the water absorption ability of a scaffold could influence the cell differentiation process in the body and could determine the interaction between the scaffold with blood and other body fluids [71].

Xu and colleagues (2016) postulated that the ideal range of WVTR for wound dressing is around 2028 gm^2^/24 h. They further concluded that, within that range, a biomaterial could maintain the moisture content, which is essential for HEKs and HDFs to multiply and exert their functions efficiently during wound re-epithelialisation. The paper also mentioned that a lower WVTR could impede wound healing due to the poor drainage of exudates and a high WVTR could cause the wound surface to dry due to a loss of fluid [72]. Furthermore, the WVTR varies depending on the type of wound and stage of healing. For instance, a granulated wound with a high amount of exudates needs a higher absorptive capacity to remove the excess fluid, while a normal skin wound and first-degree burn fall in the range of WVTR at 204 gm^2^/24 h and 276 gm^2^/24 h, respectively [73]. In fact, diabetic wounds are categorised as wounds with a high production of exudates, which often lead to tissue maceration caused by prolonged active inflammation. Therefore, CLC exhibited the highest optimal WVTR (2194 ± 66.81 gm^2^/24 h), followed by CLB (2013 ± 44.09 gm^2^/24 h), which is an ideal skin substitute for diabetic wound healing that could provide a moist environment for the surrounding wound bed [74]. CLB exhibited a lower WVTR because the bilayer scaffold made of two layers prevents water vapour molecules from passing through the layers before undergoing evaporation [35]. On top of that, it is high in hydrophobic ester groups and low in hydrophilic hydroxyl groups of CA and cellulose, which lowers the WVTR [75]. Overall, when the water contact angle increases, the hydrophobicity of the materials is also elevated. Studies revealed that a good biomaterial’s contact angle should be around 55°, which is suitable for holding more water and allowing cell attachment [18]. Moreover, Sama and colleagues reported that scaffolds with a contact angle below 90° are considered an acceptable value for cell adhesion to take place during wound healing [76]. All the groups exhibited contact angles below 90°; thus, the groups maintained a good surface wettability [49]. 

Simple mechanical tests like compression were conducted along with resilience by looking at the scaffold’s capability to regain its original shape and size after being compressed and placed in water. Our data showed that CLC and CLB (77.18 ± 1.45% and 71.22 ± 0.69%, respectively) had the highest percentage of compression to withstand the extra pressure exerted onto them and returned to original structure compared to the NC groups. For resilience, the CL groups exhibited the highest percentage of resilience with CLC (81.40 ± 1.82%), CLB (75.03 ± 1.34%), and CLG (70.03 ± 2.88%) than the NC groups. Overall, NC groups showed the lowest percentage, proving that the scaffolds cannot retain their original shapes and sizes even after being compressed and immersed in water [35]. Resilience is as equally important as porosity to maintain the elasticity and water movement in and out of the pores for cell migration [59]. On average, a good biomaterial or scaffold should fully degrade after 2 weeks of implantation in a skin wound. A degradation that takes place any faster than that could affect the mechanical properties of the scaffolds or a degradation that is too slow will provoke inflammatory reactions and the implanted scaffolds will peel off the skin [77]. The current findings show that CLC and CLB have a good biodegradation rate compared to the NC groups. Scaffolds with good biodegradation are important in tissue engineering. This is because it will permit the skin cells and tissue to regenerate followed by the subsequent degradation of the biomaterial (bilayer scaffolds) to ensure the formation of new tissue and the construction of ECM. The successful degradation of the biomaterial (bilayer scaffolds) also takes place through an inflammatory reaction by macrophages and neutrophils during the healing mechanism [78]. The idea of using collagenases is to imitate the actual conditions of the chronic wound environment, where the fabricated bilayer scaffold should resist instant degradation and maintain stability after being drenched in the mixture of collagenase. In actual chronic wound healing, MMPs, like collagenases, gelatinases, stromelysins, and others, prolong inflammation and prevent the synthesis of ECM components (collagen, elastin, laminin, and fibronectin), which are essential for wound recovery [74]. The Young’s modulus for the CL collagen and bilayer scaffolds fell in the range of 4–20 MPa, which is the ideal range for the skin [9]. A previous study pointed out that a good wound material with stability will resist as much stress from being stretched and has a lower Young’s modulus or elasticity after being stretched [73]. On the contrary, a study by Halder and colleagues stated that an increase in the Young’s modulus was essential to resist any external stress and modulate the skin cell interactions that took place during the durotaxis process in the skin [79]. On the other hand, with an increase in the concentration of crosslinking, the tensile strength also escalates, but the percentage of elongation at break declines [46]. However, in this study, with an increase in the ultimate tensile strength, the percentage of elongation at break increased for the crosslinked collagen and bilayer scaffolds, as depicted in Figure 7f. This could be explained based on the reaction of 0.1% of GNP in collagen and the intramolecular and intermolecular mechanisms of crosslinking interconnectivity between the GNP in Na-CMC with 0.5% of CA in CLB [6]. Meanwhile, CLB displayed good tensile characteristics like CLC, which is suitable for wound healing applications. The tensile strength plays a vital role in curbing the wounded area from further causing abrasion when implanting the bilayer scaffolds [52]. The bilayer scaffolds should have similar features to the human skin and the design of the scaffolds is based on the area of the wounded part. This includes maintaining stability upon insertion into the wounded site till the recovery of the wound. Other than looking into the rigid structure of the bilayer scaffolds alone, maintaining equity between the tensile strength was quite important because a tensile strength that is too high or too low will cause the accumulation of more ECM or the deterioration of the bilayer scaffolds, respectively [79]. In the present study, CLC and CLB proved to have excellent mechanical characteristics after being crosslinked with GNP [9]. Additionally, the achieved tensile strength of the collagen and bilayer scaffolds in this study was between 6 MPa and 13 MPa, which is suitable for the growth of skin cells [76]. Nonetheless, our findings are the same as those of the previous paper [9]. Therefore, we can conclude that the bilayer scaffolds could be a potent substitute for skin wound as they exhibited some mechanical features imitating the actual human skin functions [9]. 

The HEK and HDF cells’ cytocompatibility were evaluated with the collagen and bilayer scaffolds by testing the cytotoxicity and cell viability. The live and dead fluorescence assay validated the MTT cell viability assay. These tests showed no cytotoxic effects on HEKs and HDFs after being seeded on collagen and bilayer scaffolds. The HDF cells were crucial for the synthesis of ECM constituents and HEK cells to form an external layer of the skin to facilitate the reconstruction of new tissue at the wounded site [54,80,81]. In the live and dead assay, the collagen and bilayer groups exhibited good biocompatibility for HEKs and HDFs, which was related to the metabolic activity of cell mitochondria [80]. The cell viability and proliferation were quite good, and similar patterns of cell increases were observed in both cell types for the MTT test from Day 1 to Day 7. This might be due to the high number of cells that could enhance cell adhesion and multiplication, mainly supported by the collagen sponge [7]. There were no significant differences in cell viability between CLC and CLB as both cells were seeded on the collagen; thus, the scaffolds comparatively reached confluency on day 7. Few studies have reported the usage of OTC-1 to evaluate the viability of the cells, especially HEKs and HDFs in their respective fabricated scaffolds. The results conclude that OTC-1 supported the growth of cells and cell proliferation [14,35,49,59]. Several investigations have shown that bilayer scaffolds (OTC-I crosslinked with GNP) could support different types of cells regarding cell growth, viability, multiplication, and adhesion due to the well interconnected pores of appropriate size, which is essential for wound closure [15,82]. It is also evident that GNP and the biomaterial crosslinked with it induces cell expansion as well as cell differentiation [83]. 

In this study, a lower concentration of AgNPs showed some bactericidal effects against *S. aureus* and *E. coli*. The minimum inhibitory concentration (MIC) can be considered as the lowest concentration of a particular substance/solution that can inhibit bacterial growth. Researchers had reported some antibacterial activities of AgNPs synthesized from fruit and leaf extracts respectively. Even though the synthesized methods and extractions of AgNPs differed from our current study, the obtained MIC results of *S.aureus* and *E.coli* were similar with their studies [84,85]. This could further explain why Gram-positive and Gram-negative bacteria did not have the same responsiveness towards AgNPs. It might be due to the different structural membrane and cell types of bacteria [86]. In line with AgNPs, GQDs also showed some antibacterial activity based on the MIC values. According to a previous paper, GQDs became more effective in killing bacteria when using small GQDs compared to large ones because small GQDs had high oxidative stress and achieved their killing efficiency by increasing the permeability of the bacterial cell [32]. Moreover, GQDs become potent antibacterial agents when they are developed as functionalise GQDs or conjugated with other polymers [31].

## 5. Conclusions

In this study, the crosslinked bilayer scaffolds were compared with crosslinked collagen (CLC). The data demonstrated that there were not many differences between CLC and CLB in terms of physical, chemical, and mechanical properties through the freeze-drying method; when crosslinked with GNP, they showed good physical, chemical, and mechanical properties. Based on the results, we can infer that collagen modified with gauze is ideal to serve its purpose as an acellular skin substitute for treating skin wounds, particularly infected wounds. The presence of CA maintains the stability of Na-CMC gauze, while the tested nanoparticles’ concentrations possess some antimicrobial activities. The constructed bilayer scaffolds are also highly biocompatible with HEKs and HDFs cells. The idea of this study is that the fabricated bilayer scaffold should be functionalised like normal human skin to regenerate the diabetic wound within 14 days without microbial infections and scar formation. To achieve this, the experiments should be repeated with nanoparticle-coated gauze to determine the physicochemical and mechanical properties as well as in a cell study. If the fabricated bilayer scaffolds (Na-CMC with collagen) can be utilised for diabetic wounds, and then, this bilayer scaffold can be used in other wounds, such as acute, burn, and pressure wounds. Most of these wounds are at risk of infection if there are delays in treatments. Therefore, scaffolds with nanoparticles are highly recommended. Some modifications are needed for the bilayer scaffolds to suit the type/nature of the wounds, such as the increase in the concentrations of nanoparticles; combinations of nanoparticles (AgNPs with GQDs) to enhance the antibacterial effects for larger wounds or deeper wounds; increase in the concentration of GNP, Na-CMC, and CA; and fabrication of larger collagen scaffolds so that the scaffolds can cover the wounds, absorb more exudate, and provide a moist environment. There are some limitations of the study such as the results generated in the current study are from in vitro tests. A further investigation using an in vivo validation of the fabricated scaffolds is required to study the effect of bilayer scaffolds on diabetic mice. Furthermore, more bacteria with different strains should be included in the study to determine the bacterial effects against the nanoparticles and integrated gauze with nanoparticles. The current fabricated scaffold is limited to wound management. To study drug delivery systems, we need different modifications of the gauze and to combine the tested drug with nanoparticles, e.g., GQDs and liposomes, to see the effects on pathogens. 

## Figures and Tables

**Table 1:** Weight loss in % for the CL and NC groups and degradation steps from 1st weight loss to 4th weight loss.

Samples	1st Weight Loss	2nd Weight Loss	3rd Weight Loss	4th Weight Loss
	Initial Temperature	Volatile	Decomposition	Combustion
	30 ℃	(180–300 ℃)	(350–400 ℃)	(490–593 ℃)
NCG	98%	86.61%	34.11%	26.52%
CLG	99.93%	84.91%	41.01%	30.58%
NCC	99.90%	83.31%	36.66%	20.13%
CLC	100.07%	85.88%	55.76%	43.65%
NCB	9978%	84.09%	36.10%	24.65%
CLB	99.87%	66.11%	45.64%	38.45%

**Table 2:** EDX study for both crosslinked and non-crosslinked groups. Carbon, nitrogen, and oxygen are the major elements present in all the groups.

Samples	Elements (%)			
	C	N	O	Na
NCG	40.59 ± 0.82	1.10 ± 0.07	58.10 ± 0.29	9.30 ± 0.90
CLG	37.80 ± 0.67	1.40 ± 0.23	51.60 ± 0.34	7.00 ± 0.24
NCC	58.04 ± 0.09	19.50 ± 0.10	34.10 ± 0.57	
CLC	48.50 ± 0.23	16.50 ± 0.12	25.40 ± 0.08	
NCB	41.40 ± 0.83	2.59 ± 0.02	50.60 ± 0.51	14.2 ± 0.19
CLB	36.10 ± 0.43	1.56 ± 0.11	48.20 ± 0.33	6.50 ± 0.24

**Table 3:** Minimum inhibitory concentration of AgNPs, GQDs, and gentamicin against *S. aureus* and *E. coli*.

Antibacterial Agent	*S. aureus*	*E. coli*
AgNPs	0.25	0.125
GQDs	0.5	0.5
Gentamicin	0.0625	0.0625

## Data Availability

All data are available within this article.
